# Clinicopathological characteristics and outcomes of 23 patients with secretory carcinoma of major salivary glands

**DOI:** 10.1038/s41598-021-01970-4

**Published:** 2021-11-22

**Authors:** Jingjing Sun, Sirui Liu, Kun Fu, Ning Gao, Rui Li, Wei He, Zhenjie Gao

**Affiliations:** Department of Oral and Maxillofacial Surgery, The First Affiliated Hospital of Zhengzhou University, Jian She Road, Zhengzhou, 450052 China

**Keywords:** Oncology, Signs and symptoms

## Abstract

This retrospective study investigated the clinicopathological characteristics of secretory carcinoma of salivary glands (SCSG) in 23 patients with histopathologically confirmed SCSG between January 2010 and December 2020. In total, 13 males and 10 females (ratio, 1.3:1) aged 10 − 69 years (median, 45 years) were enrolled in this study; the average disease duration was 2.44 years (0.25–20 years). Twenty-one patients (91.3%) had SCSG in the parotid gland, and two (8.7%) in the submandibular gland. All patients had single nodules of diameters 0.8–4.8 cm (average 2.6 cm); five with lymph node metastases, and two with distant metastases. Immunohistochemically, tumors stained positive for S-100, mammaglobin, CK7, GATA3 and pan-Trk, and negative for DOG1, P63, and calponin, with Ki-67 positivity from 1 to 50%. *ETV6* gene rearrangement was confirmed in 15 patients. All patients underwent oncological resection, four had radioactive particles implanted postoperatively, one received chemotherapy, and seven underwent chemoradiotherapy. Six patients had regional recurrences, two distant metastases, and one died before the last follow-up. SCSGs are typically indolent, with a low locoregional recurrence rate and excellent survival. Prognosis is correlated to clinical stage, pathological grade, and surgical procedures.

## Introduction

Diagnostic criteria for secretory carcinoma of salivary glands (SCSG), a recently described rare malignant tumor, were first introduced by Skálová et al.^1^. Although these tumors were originally classified as mammary analog secretory carcinomas of the salivary glands, their histomorphological and immunohistochemical features are similar to those of secretory carcinoma of the breast^2^. Accordingly, the updated World Health Organization (WHO) Classification for Head and Neck Tumors (4th edition, 2017) substituted mammary analog secretory carcinoma with secretory carcinoma^3^.

These salivary gland tumors are characterized by the presence of a t(12;15)(p13;q25) translocation, which leads to the fusion of the translocation-Ets-leukemia virus (*ETV6*) gene on chromosome 12, and the neurotrophic tropomyosin receptor kinase 3 (*NTRK3*) gene on chromosome 15^4^. Before the introduction of SCSG, these tumors were primarily diagnosed as acinic cell carcinoma (AciCC), mucoepidermoid carcinoma, adenocarcinoma, or not otherwise specified^5^.

The NCCN Clinical Practice Guidelines in Oncology (Head and Neck Cancers, Version 2.2020) and ASCO Guidelines (Management of Salivary Gland Malignancy) on the diagnosis and treatment of SCSG with *NTRK* alterations have been revised^6,7^. Before the revision, research on the clinical characteristics, treatment, and outcomes of this tumor was limited^8^. Here, we present a review of 23 patients with SCSG in major salivary glands who were treated in our department at the First Affiliated Hospital of Zhengzhou University, including a detailed discussion of clinicopathological characteristics, outcomes of treatment, and prognosis.

## Materials and methods

This retrospective study, the protocol for which was approved by the Zhengzhou University Hospital Medical Ethics Committee (Ethics Review Number: 2020-KY-230), was conducted in accordance with the principles of the Declaration of Helsinki. All participants and parents/legal guardians of the participants < 16 years of age provided signed informed consent.

We retrospectively enrolled 23 patients with histopathologically confirmed SCSG who had been treated between January 2010 and December 2020 at the First Affiliated Hospital of Zhengzhou University. SCSG diagnosis was blindly reviewed by three independent consultant pathologists, all experts in salivary gland pathology.

For conventional microscopy, formalin-fixed, paraffin-embedded tissues were stained with hematoxylin and eosin. Selected immunohistochemical stains were used in all cases, including S-100 (ZA-0225, ZSGB-BIO, Beijing, China), mammaglobin (ZM-0388, ZSGB-BIO), CK7 (ZM-0071, ZSGB-BIO), GATA3 (ZA-0661, ZSGB-BIO), DOG1 (ZM-0371, ZSGB-BIO), P63 (ZM-0406, ZSGB-BIO), calponin (ZA-0524, ZSGB-BIO), and Ki-67 (ZM-0166, ZSGB-BIO). Pan-Trk immunohistochemistry (Pan-Trk IHC) was performed using a pan-Trk rabbit monoclonal antibody (EPR17341, Roche). Pan-Trk IHC was considered positive using following criteria: (1) any staining (cytoplasmic or nuclear) within the tumor cells, and (2) any nuclear staining in the tumor cells. Fluorescence in situ hybridization (FISH) was performed to detect *ETV6* using the Dual Color Breakapart probe (04N09-020, ZSGB-BIO) according to the manufacturer’s protocol. Tumor cell nuclei were examined for the presence of colocated (yellow) or translocated (green and red) signals.

Patient clinical data were analyzed for sex, age, tumor size, symptoms, and their duration, means of diagnosis, TNM stage, treatment administered, recurrence, and prognosis.

## Results

### Clinical characteristics

The 23 patients with SCSG comprised 13 males and 10 females (1.3:1). The median age was 45 years (range 10–69 years) and the average duration of disease from initial symptoms to diagnosis was 2.44 years (range 3–20 years). Twenty-one of the tumors (91.3%) were located in the parotid gland (four left parotid, 17 right parotid) and two (8.7%) in the submandibular gland. All 23 patients had single nodules presenting as painless masses of diameters 0.8–4.8 cm (average 2.6 cm). Six patients (26.1%) had T1 tumors, 14 (60.9%) had T2 tumors, and 3 (13%) had T3 tumors. Five patients presented with regional lymph node metastases, and two had distant metastases at the time of diagnosis (Table 1).

**Table 1:** Patient and tumor characteristics (n = 23).

Characteristic	Value
Sex, n (%)	
Male	13 (56.5%)
Female	10 (43.5%)
Median age, in years [range]	45 (10–69)
Tumor localization, n (%)	
Major salivary glands, n (left, right)	
Parotid	21 (91.3%, 4, 17)
Submandibular gland	2 (8.7%, 1, 1)
Tumor characteristics	
Size (cm)	2.6 (0.8–4.8)
Texture	Firm
Boundary	Well-demarcated
Fixation	Elastic and mobile
Signs and symptoms	Painless and no facial paralysis
TNM stage, n	
T1/T2/T3/T4/Tx	6/14/3/0/0
N0/N1/N2/N3	18/1/4/0
M0/M1	21/2

**Figure 1:**
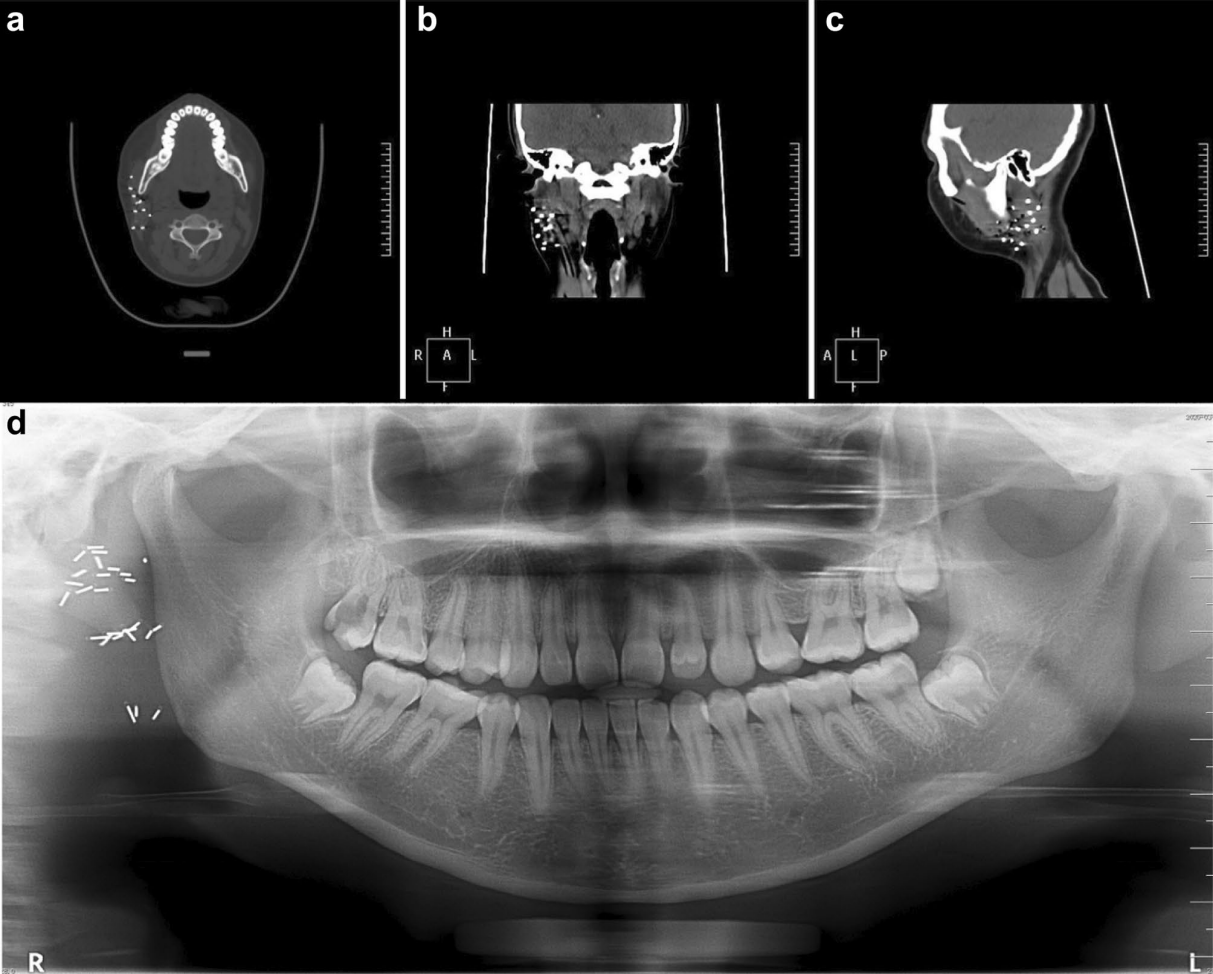
A typical postoperative implantation of I^125^ radioactive particles. (**a**) Horizontal plane; (**b**) coronal plane; (**c**) sagittal plane; (**d**) panoramic radiography.

All 23 patients underwent surgery as the primary treatment. The affected salivary glands were resected from 14 patients, seven patients underwent local excision, and the precise extent of surgery for the remaining two patients could not be determined. None of the initial surgeries included neck dissection. Details of the treatment administered, and the outcomes are summarized in Table 2. One of the 11 patients who underwent only surgical intervention presented with local recurrence 42 months after the primary surgery. Moreover, none of the four patients who underwent postoperative implantation of I^125^ radioactive particles under magnetic resonance imaging (MRI) navigation (average of 35 particles) presented with local recurrence; a typical case is shown in Fig. 1. One patient who received chemotherapy had a local recurrence, and four of the seven who underwent chemoradiotherapy had local recurrences. Five patients developed lymphatic metastases (21.7%), two developed distant metastases (8.7%), and one patient died during follow-up (4.3%).

**Table 2:** Outcomes of 23 patients with SCSG treated by different therapies.

Treatment	Cases	Recurrence	Lymphatic metastasis	Distant metastasis	Death
Operation only	11	1	0	0	0
Operation + I^125^	4	0	1	0	0
Operation + chemotherapy	1	1	1	0	0
Operation + chemoradiotherapy	7	4	3	2	1
Total	23	6	5	2	1

SCSG, secretory carcinoma of salivary gland.

### Imaging findings

**Figure 2:**
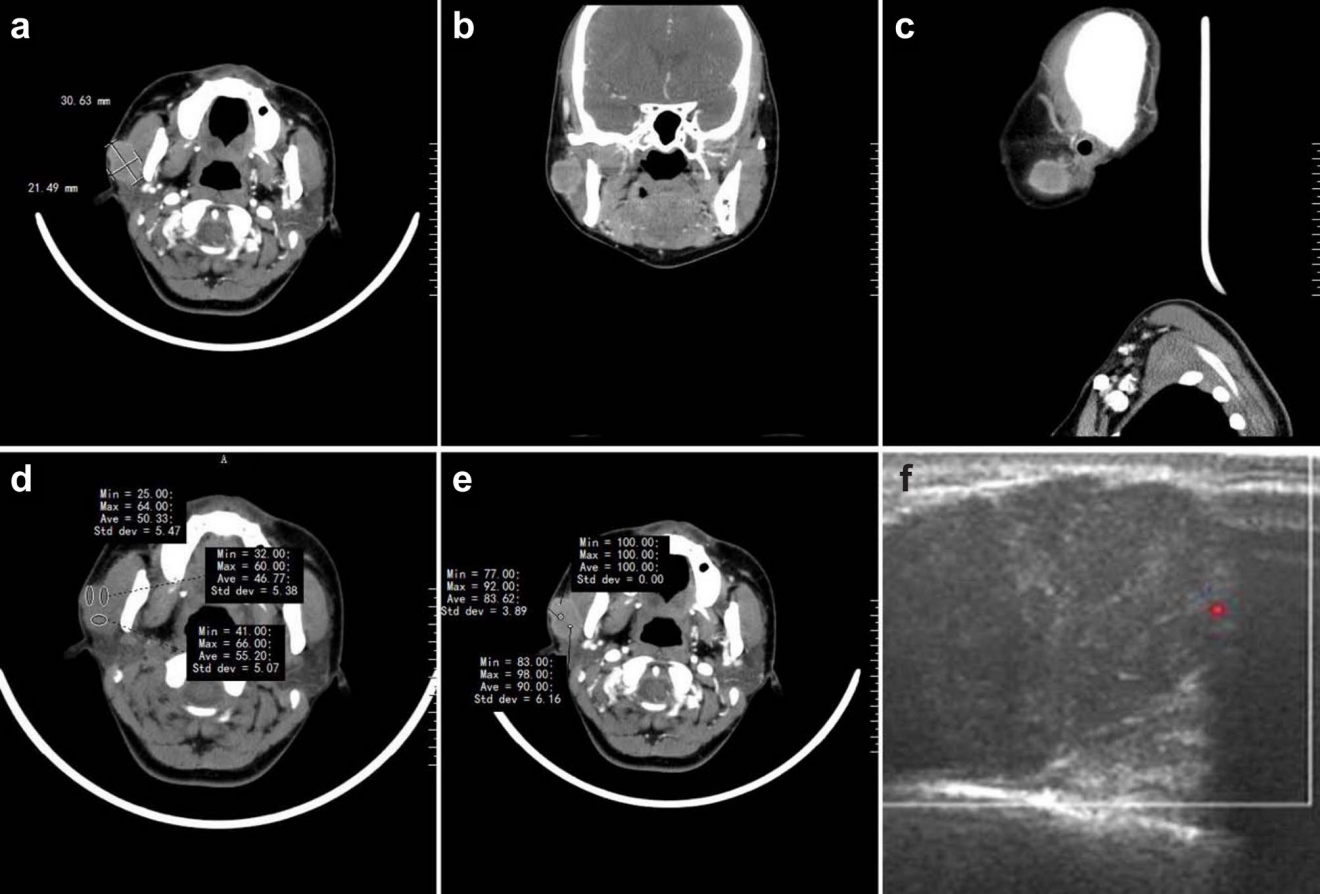
Color Doppler ultrasound and computed tomography (CT) imaging findings of typical SCSG. In CT, the lesions appeared oval or lobulated, with clear boundaries, regular edges, and uneven density. (**a**) Horizontal plane; (**b**) coronal plane; (**c**) sagittal plane. (**d**) Average Hounsfield unit (HU) was 42 (41.69 ± 16.5) with plain scanning, and (**e**) 76 (76.1 ± 24.7) with enhanced scanning. (**f**) Typical ultrasonic characteristics.

On computed tomography (CT), the lesions appeared oval or lobulated, with clear boundaries, regular edges, and uneven density. Some had low-density cystic areas and calcification foci (Fig. 2a–c). In plain scanning, the average Hounsfield unit (HU) was 42 (41.69 ± 16.5), and 76 (76.1 ± 24.7) with enhanced scanning (Fig. 2d,e). The degree of enhancement was primarily uneven and marked (> 40 HU); however, a few tumors showed mild enhancement (10–20 HU), or no obvious enhancement (< 10 HU). Most recurrences showed marked enhancement. Slightly enlarged lymph nodes were found in the ipsilateral submaxillary region and carotid sheath, potentially resulting in a misdiagnosis of pleomorphic adenoma.

Color Doppler ultrasound revealed that the tumors were well-demarcated, heterogeneous, and hypoechoic masses with regular morphology. Most tumors were solid, although some appeared as a cystic and solid combination. Punctate blood flow signals were observed within the lesions, which can readily lead to the misdiagnosis of pleomorphic adenoma. Typical ultrasonic characteristics are shown in Fig. 2f.

**Figure 3:**
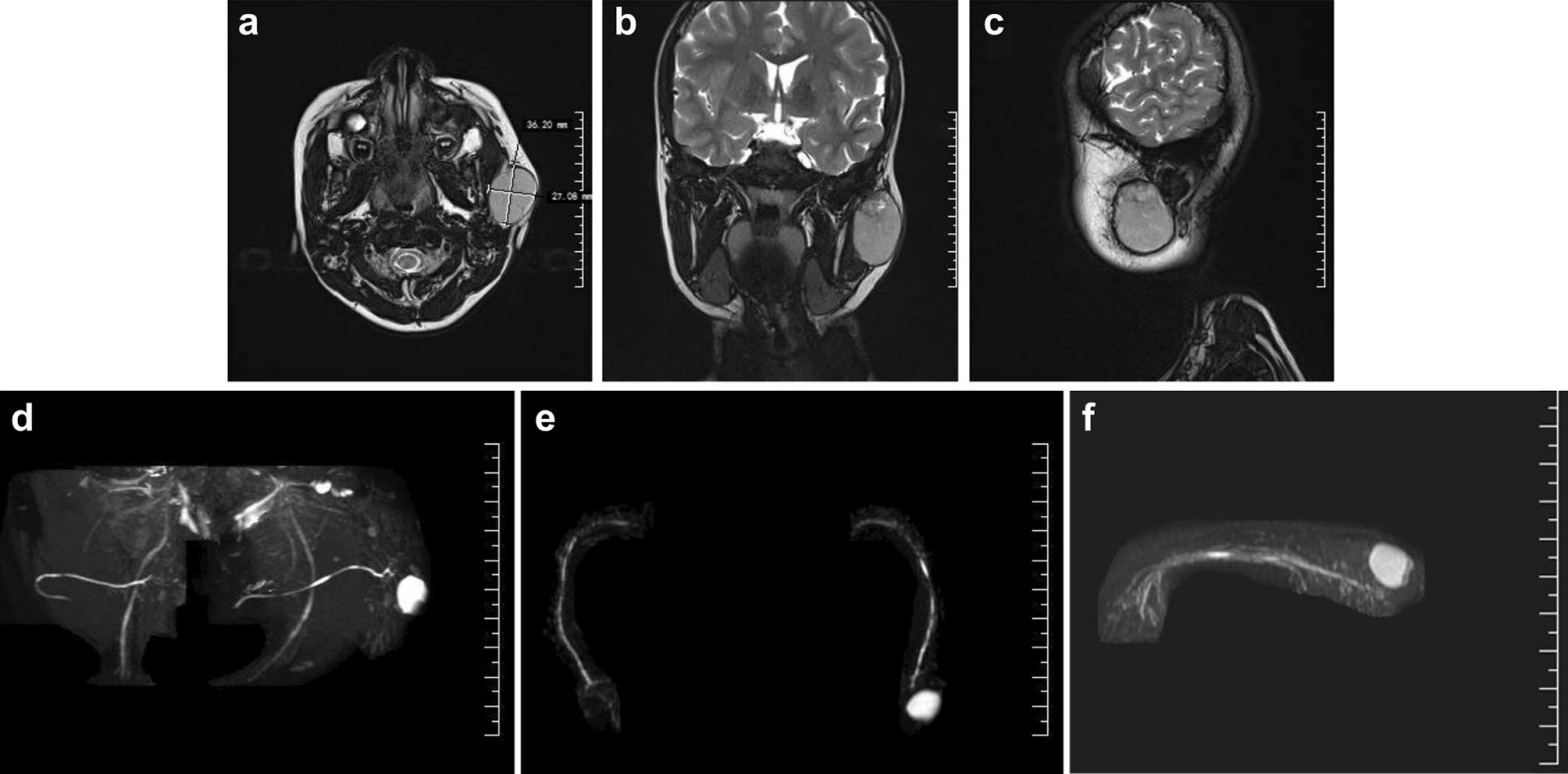
Magnetic resonance imaging (MRI) imaging findings of typical SCSG. On MRI, tumors tend to be roundish in shape, with clear boundaries. T2 images showed long signals or mixed long and short signals; (**a**) horizontal plane; (**b**) coronal plane (**c**) sagittal plane. MRH detected lesions located in the posterior lower pole of the parotid gland; (**d**) coronal plane (**e**) horizontal plane (**f**) sagittal plane.

Using MRI, we found that SCSGs consistently had the following characteristics: (1) a tendency to be round with clear boundaries; (2) most T1 or T2 images had long signals or mixed long and short signals. The fat-suppression images showed high signals. On diffusion-weighted imaging, minimal high b-value diffusion was observed, with high signals and uneven enhancement ranging from slight to significant. As with the CT findings, slightly enlarged lymph nodes were found in the ipsilateral submaxillary region and carotid sheath (Fig. 3a–c).

Moreover, two patients underwent magnetic resonance hydrography (MRH); in both patients, lesions were observed in the posterior lower pole of the parotid gland, more than 3 mm away from the main duct, which appeared normal. Regional parotid gland resection was therefore performed to preserve the secretory function of the parotid duct and a portion of the gland (Fig. 3d–f).

### Pathological characteristics

**Figure 4:**
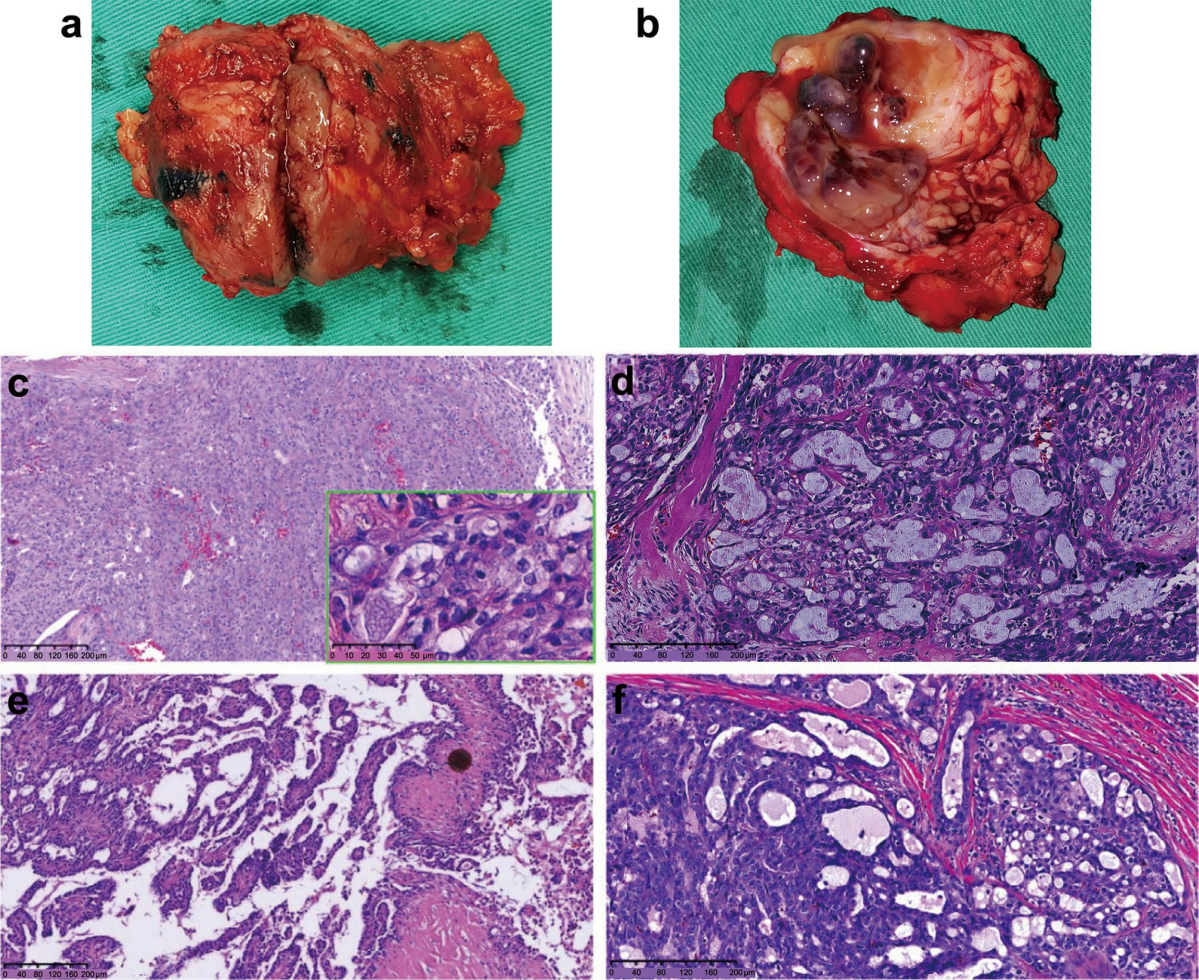
Pathological characteristics. (**a**) An isolated solid nodule tumor with light-tan or grayish cut surface. (**b**) Tumor-harboring cysts containing yellowish-white fluid; (**c**) solid structures; (**d**) microcystic structures; (**e**) follicular and papillary cystic structures; (**f**) tubular structures. At high magnification, the tumor cells are round or oval with little atypia. They have eosinophilic granular or vacuolated cytoplasm with small, uniform nuclei (green box).

Grossly, the tumors were isolated as solid nodules with light-tan or grayish cut surfaces (Fig. 4a). Their texture was hard, with clear boundaries and an average diameter of 2.6 cm (0.8–4.8 cm). A few tumors harbored cysts containing yellowish-white fluid (Fig. 4b).

The tumors exhibited a lobulated growth pattern with fibrous septa and were composed of microcystic/solid, tubular, follicular, and papillary cystic structures with distinctive luminal secretions (Fig. 4c–f). At high magnification, the tumor cells were round or oval with little atypia and had eosinophilic granular or vacuolated cytoplasm with small, uniform nuclei (Fig. 4c, green box).

**Figure 5:**
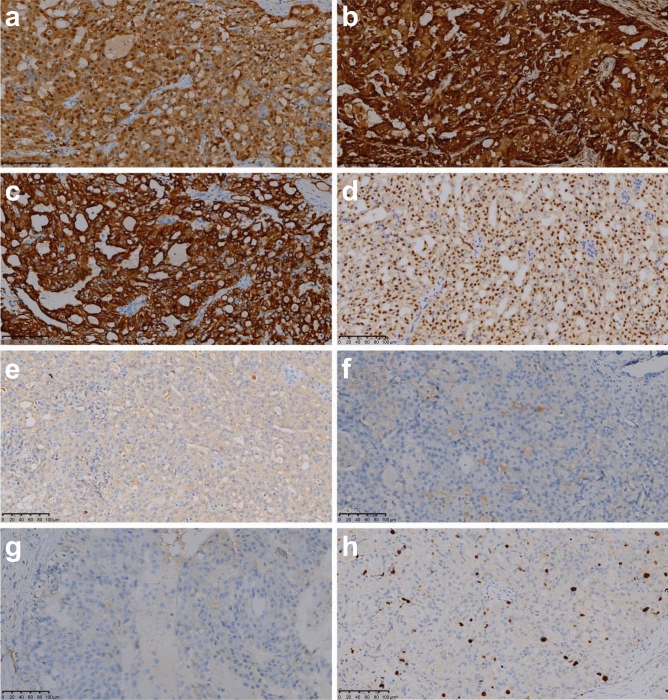
Immunohistochemical staining. S-100 (**a**), mammaglobin (**b**), CK7 (**c**), and GATA3 (**d**) were stong positivity; staining is negative for calponin (**e**), P63 (**f**), and DOG1 (**g**). (**h**) Ki-67( +) was 5%.

Furthermore, immunohistochemical staining showed strong positivity for S-100 (Fig. 5a), mammaglobin (Fig. 5b), CK7 (Fig. 5c), and GATA3 (Fig. 5d), whereas staining was negative for calponin (Fig. 5e), P63 (Fig. 5f), and DOG1 (Fig. 5g). Ki-67 positivity (Fig. 5h) ranged from 1 to 50%; it was less than 10% in 14/23 and less than 20% in (14 + 5)/23 patients.

**Figure 6:**
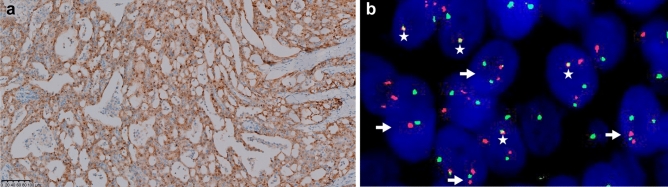
Pan-Trk IHC and FISH of *ETV6* . (**a**) Pan-Trk IHC showing nuclear and/or cytoplasmic staining, (**b**) *ETV6* gene rearrangement confirmed by fluorescence in situ hybridization as tumor cells with split signals (arrows) adjacent to *NTRK3* (stars).

Pan-Trk IHC in our cohort was positive exhibiting cytoplasmic and/or nuclear staining (Fig. 6a). Finally, FISH data were available for 15 patients (Fig. 6b). These patients, who had not been definitively diagnosed by immunohistochemistry, were all diagnosed on the basis of *ETV6* gene rearrangement.

## Discussion

SCSGs are generally low-grade salivary gland carcinomas characterized by morphological resemblance to mammary analog secretory carcinoma and *ETV6–NTRK3* gene fusion^2,9^. In 2017, SCSGs were added to the WHO classification of head and neck tumors^3^.

The age at onset of SCSG reportedly varies widely. Although these tumors generally develop in adults, they can also occur in children and adolescents; however, no apparent sex-based bias has been reported^10^. In the present study group, the median age was 45 years (range: 10–69 years) with a male:female ratio of 1.3:1. Moreover, the parotid gland represents the most common site of SCSG, although small salivary glands, including the buccal, upper lip, and palatal glands, can also be involved^11–13^. Indeed, the tumors of 21 patients in the current study were located in the parotid gland, those of 17 in the right parotid gland, and those of 4 in the left parotid gland, differing only slightly from previously reported findings^14^. SCSGs most commonly present as painless, slow-growing, well-circumscribed masses that are often misdiagnosed as pleomorphic adenoma or acinar cell carcinoma^15^. Consistent with the present study, most patients presented with a painless mass, with only a few recurrences sensitive to touch. Moreover, no specific preoperative ultrasonography, CT, or MRI findings were identified. The tumors presented as well-demarcated masses of uneven density, sometimes with cystic changes that could easily be misdiagnosed as pleomorphic adenomas. Additionally, only three patients presented with T3 tumors, whereas the others had T1 or T2 tumors. Moreover, T4 tumors have been reported by another study^14^.

Before their official characterization, SCSGs were frequently classified as AciCCs owing to their nearly identical histological growth patterns^16^. Although no significant clinical differences have been described between SCSGs and AciCCs, they may exhibit considerable histopathological morphological differences. For instance, unlike AciCCs, SCSGs have no secretory zymogen cytoplasmic granules that undergo true positive periodic acid–Schiff reactions^17^. Additionally, the nuclei of SCSGs have prominent pseudo-inclusion bodies; however, SCSGs do not contain other cell types, such as serous, intermediate, and clear cells, which are commonly observed in AciCCs^18^. As for immunophenotypes, SCSGs can express various markers of breast secretory carcinomas, including mammaglobin, S-100 protein, STAT5A, MUC1, MUC4, GCDFP-15, CK7, CK8, CK18, CK19, and epithelial cell membrane proteins^19^. However, they characteristically do not express DOG-1, estrogen, androgen, progesterone receptors, or HER2^20^. In contrast to SCSGs, AciCCs do not express mammaglobin, or exhibit only weak local expression; however, they express DOG-1 diffusely^20^. Khalele et al. reported that SCSG can be diagnosed via the detection of mammaglobin and S-100 protein in the salivary gland tumor, in the absence of DOG-1^21^. It has also been reported that if mammaglobin and S-100 are strongly positive, SCSG can be diagnosed without resorting to molecular biological methods^22^, making mammaglobin a particularly important tumor marker for SCSG diagnosis.

Similar to secretory carcinomas of the breast, SCSGs have been shown to contain the translocation t(12;15) (p13;q25), resulting in *ETV6*–*NTRK3* fusion^4^, which has not been found in other types of salivary gland tumors. However, this has been reported in congenital fibrosarcoma, mesodermal renal tumor, and acute myeloid leukemia^23,24^. The *ETV6–NTRK3* fusion gene produces a chimeric tyrosine kinase that activates two major effector pathways—namely, the Ras/mitogen-activated protein kinase (MAPK) and phosphoinositide 3-kinase/Akt pathways, both of which seem to be required for *ETV6-NTRK3* transformation^25^. The detection of *ETV6–NTRK3* fusion is important because targeted therapies are directed against tropomyosin receptor kinases (Trk). Various techniques have been used to detect *ETV6–NRTK3* fusion, including FISH, reverse transcription polymerase chain reaction (to detect the *ETV6–NTRK3* fusion transcript), next-generation sequencing, and Pan-Trk IHC^26^. In the present study, 15 patients who had not been definitively diagnosed using IHC were all diagnosed on the basis of the *ETV6* gene rearrangement detected in the current study. Additionally, Pan-Trk IHC results were positive. Prior to IHC and *ETV6* gene detection in the current study, four of the 23 patients’ tumors were misdiagnosed as acinar cell carcinomas, three as mucoepidermoid carcinomas, two as adenoid cystic carcinomas, one as low-grade ductal carcinoma, and one as squamous cell carcinoma. In addition, recent findings have expanded the molecular profile of SCSG to include multiple novel *ETV6* fusion partners, including *ETV6-RET* and *EGFR–SEPT14*^27^*.* Gene therapy may offer a new hope to patients with this tumor type in the future^28^.

SCSGs behave similarly to AciCCs; therefore, the treatment of SCSGs is primarily centered on surgical interventions. Although secretory carcinoma is still classified as a low-aggression salivary gland carcinoma by the 2021 ASCO Guidelines, secretory carcinoma of the salivary glands with high-grade (HG) transformation has been reported^24,29^. Hence, currently, there is no consensus for the optimal treatment of these tumors. Some researchers believe that SCSGs are indolent tumors that rarely cause distant metastases, making surgery alone a sufficient therapeutic strategy^30^. Moreover, another study has indicated that surgery combined with postoperative radiotherapy is more effective^31^. Until 2020, the NCCN guidelines recommended *NTRK* therapy options such as larotrectinib and entrectinib for patients with recurrent *NTRK* fusion-positive salivary gland tumors and distant metastases, and they are considered new strategies for SC treatment^32,33^. In the present study, 11 patients were treated with surgery alone, one of whom had postoperative recurrence. In addition, one patient received postoperative chemotherapy and later had a recurrence, whereas four of the seven patients who received postoperative chemoradiotherapy had recurrences. The overall recurrence rate was 26.1%.

Interestingly, six patients with recurrences underwent their first surgeries in local primary hospitals, and their recurrences were considered to have resulted from incomplete resection after the first surgery. Although, currently, few reports are available on the different surgical procedures for SCSGs, some advocate for the simultaneous total resection of the affected lobe of the parotid gland and cervical lymph node dissection^34^. Indeed, Williams and Chiosea ^35^ reported a higher incidence of lymph node metastases from SCSGs than from AciCCs (33% vs. 8%); however, cervical dissection is still rarely performed^36^. In the current study, five patients developed cervical lymphatic metastases, and two developed distant metastases (both to the lung) after local recurrences. All six patients with local recurrences only underwent tumor enucleation or partial parotid gland resection; none of the patients who underwent excision of the tumor or the superficial lobe of the parotid gland had recurrences or distant metastases. Taken together, these results suggest that preoperative determination of the size and extent of the tumor is vital to successful surgical interventions.

Recently, MRH of salivary gland ducts has been shown to clearly establish the three-dimensional relationship between the tumor and parotid duct, while also detecting dilation, stenosis, displacement, and destruction of main ducts and branch ducts, potentially guiding preoperative planning^3^. For instance, if the tumor has invaded the main duct, retaining the gland is meaningless; instead, superficial or total parotid gland resection should be performed where possible. Moreover, if the tumor is sufficiently distant from the main duct, regional parotid gland resection with preservation of a portion of the gland can be considered. Thus, detailed MRH can accurately guide the planning of parotid gland preservation surgery, potentially enabling the retention of some functional glands, while completely resecting the tumor. In the present study, two patients underwent functional preservation surgery with the precise guidance of MRH. Most of the parotid glands and dominant ducts with secretory function were retained, and the probability of facial nerve injury during surgery was greatly reduced. Their postoperative function and appearance were significantly improved compared with those in other patients. For example, surgical scar was small, local facial collapse was not obvious, and postoperative facial symmetry was improved in these two patients. No obvious facial paralysis occurred in the two patients, and the patients were satisfied with the surgical effect after surgery. In addition, no other treatment was performed in the two patients after surgery, and no local recurrence or distant metastasis was found in the follow-ups. MRH before surgery is beneficial to minimally invasive surgery and functional surgery for parotid gland malignant tumors^37^.

The prognosis of SCSG is related not only to the first operative procedure but also to age, clinical stage, and Ki-67 proliferation index. Although six of the 23 patients in this study had local recurrences (mean age 53 years) and one patient died, the overall prognosis was good, which is consistent with previously reported results^38^. Additionally, children and young patients generally have better prognoses, whereas adult patients have more aggressive tumors, with older patients (more than 60 years) having the most aggressive tumors and the highest recurrence rates. This may be related to differences in pathological features at different ages and the characteristically poor immune function of older patients^39^. In addition, prognosis becomes worse with increasing clinical stage and HG transformation^40^. HG-transformed SCSG is a considerably more aggressive tumor that follows an accelerated clinical course, resulting in local recurrences, cancer dissemination, and death^29^. Nevertheless, in the present study, microscopic findings were identical in all patients, and none of the neoplasms were composed of HG components; this may have resulted from the limited cases. Furthermore, lymph node involvement and extra-parenchymal glandular invasion are associated with a greater risk of local recurrence and metastasis^41^. In the present study, five patients developed lymph node metastases, all of whom underwent therapeutic neck dissection and postoperative radiotherapy and/or chemotherapy. However, they all experienced postoperative recurrences, with pulmonary metastases detected in two patients, one of whom died. Additionally, the tumor of the patient who died had a Ki-67 proliferation index of 50% + and had recurred three times. This is consistent with reports that patients with a Ki-67 proliferation index of more than 10% have poor prognoses^42^.

In summary, SCSG is a rare type of low-grade malignant salivary gland tumor that commonly occurs in the parotid gland, rarely invades the surrounding tissues, and has a good prognosis. Moreover, histomorphological and immunohistochemical characteristics are key to distinguishing SCSGs from other salivary gland tumors, with the detection of *ETV6* translocation considered the gold standard for diagnosis. Surgical resection is the main treatment, and the success of the first operation represents the major determinant of prognosis. Considering the low rate of cervical lymphatic metastasis, functional excision of SCSGs with facial nerve preservation is generally performed, depending on preoperative MRH findings concerning salivary gland duct status. Postoperative I^125^ implantation or local radiotherapy can be performed to reduce the recurrence rate. If distant metastases occur, neck dissection and postoperative treatment are necessary.

Considering that SCSG is a relatively newly described tumor type, few clinical findings have been reported, resulting in only a small number of patients included in this study cohort. Thus, definitively ascertaining the optimal treatment and outcomes of SCSG requires studies to be performed with a larger patient cohort and long-term follow-up.

## Data Availability

All data generated or analyzed during this study are included in this published article.
